# Supplementary material for: Theoretical-Computational Modeling of Gas-State Thermodynamics in Flexible Molecular Systems: Ionic Liquids in the Gas Phase as a Case Study
Source: Molecules. 2022 Nov 14;27(22):7863. doi: 10.3390/molecules27227863 (PMC9694092; doi:10.3390/molecules27227863)
Supplement: Supplementary file 1 [file molecules-27-07863-s001.zip › molecules-1993574-supplementary.pdf]

Supplementary Information for
Theoretical-computational modeling of gas-state thermodynamics in flexible molecular systems.

Andrea Amadei[1], Andrea Ciccioli[2], Antonello Filippi[3], Caterina Fraschetti[3], Massimiliano Aschi[4].

[1] Dipartimento di Scienze e Tecnologie Chimiche, Universit\`a di Roma "Tor Vergata", via della Ricerca Scientifica 1, 00133 Roma, Italia. E-mail: andrea.amadei@uniroma2.it

[2] Dipartimento di Chimica Università di Roma, "La Sapienza", P.le A. Moro 5, 00185, Roma, Italia.

[3] Dipartimento di Chimica e Tecnologie del Farmaco, Università di Roma, "La Sapienza", P.le A. Moro 5, 00185, Roma, Italia.

[4] Dipartimento di Scienze Fisiche e Chimiche Università de l'Aquila, via Vetoio (Coppito 2), 67010 l'Aquila, Italia. E-mail: massimiliano.aschi@univaq.it \\

CARTESIAN COORDINATES AND HARMONIC FREQUENCIES FOR THE REFERENCE STRUCTURES.

A. n-Butane

C	-0.001648	-0.000290	-0.002466
C	0.000388	0.004657	1.527897
C	1.407854	0.002101	2.123825
C	1.410962	0.010600	3.653440
H	0.508555	-0.888929	-0.393765
H	-1.022625	0.003105	-0.400676
H	0.515653	0.882155	-0.398114
H	-0.550569	-0.871304	1.895722
H	-0.543947	0.887842	1.889673
H	1.959296	0.876652	1.752529
H	1.952352	-0.881245	1.762793
H	0.902868	0.901151	4.042808
H	0.895586	-0.870688	4.053283
H	2.432262	0.010444	4.051563

79.3188	208.3782	241.5775
270.9190	435.0218	727.6941
818.7353	862.4816	971.9417
997.9461	1039.5837	1088.4457
1187.8102	1217.1879	1304.9826
1336.8318	1346.1739	1419.5738
1442.7100	1444.8725	1508.2351
1511.8804	1526.3355	1528.3633
1528.9665	1530.8861	3047.5502
3053.9091	3057.4988	3059.5429
3076.0776	3097.8843	3130.9193
3133.2365	3135.3277	3135.9010

B-Bmim-Ntf2

H	0.719022	-2.602775	3.938409

C	0.462336	-1.828495	3.213095
H	0.777268	-0.850852	3.574952
H	-0.611578	-1.806997	3.019428
N	1.146791	-2.106589	1.953663
C	1.079055	-3.284653	1.246568
H	0.543278	-4.142071	1.620230
C	1.732976	-3.080196	0.073699
H	1.873302	-3.725634	-0.778168
N	2.191754	-1.783315	0.081812
C	1.818402	-1.214657	1.227143
H	1.986045	-0.188827	1.513157
C	2.773457	-1.078546	-1.070606
H	3.190292	-1.852380	-1.721381
H	1.943009	-0.589354	-1.587616
C	3.843152	-0.071290	-0.672875
H	3.398014	0.702236	-0.037320
H	4.632829	-0.572939	-0.097043
C	4.442069	0.604627	-1.907847
H	4.884550	-0.155185	-2.566458
H	3.636123	1.085985	-2.475425
C	5.499411	1.644138	-1.540267
H	6.327800	1.187687	-0.985388
H	5.915794	2.115983	-2.435977
H	5.069155	2.434010	-0.914373
F	-4.120687	-0.583635	-0.562882
C	-3.097284	-1.005394	-1.297688
F	-3.013260	-0.266125	-2.398751
F	-3.311910	-2.276102	-1.647507
S	-1.506919	-0.941449	-0.330800
O	-0.458859	-1.382092	-1.251368
O	-1.729734	-1.779213	0.847377
N	-1.512004	0.624716	-0.032063
S	-0.257322	1.384680	0.573066
O	0.928992	1.417432	-0.286673
O	0.007556	1.093764	1.986444
C	-0.968837	3.104650	0.532684
F	-1.260401	3.467483	-0.711561
F	-0.046890	3.937507	1.020556
F	-2.061839	3.185930	1.282716

14.0705	22.0231	28.2414
30.2306	38.2946	49.0133
53.7311	55.4921	60.9179
73.1392	81.9690	89.0281
101.6357	122.6548	126.0593
133.0437	156.0889	186.1345
200.6493	209.3442	221.7297
248.9035	255.2858	274.9758
276.6769	284.7572	292.8033
319.2194	326.0345	331.1838
345.1022	395.6511	410.6019
477.2316	494.6744	497.9458

525.4832	543.9042	558.0150
569.2996	570.6607	581.1723
632.7245	637.3903	667.9065
677.1605	740.1096	746.6574
755.0890	774.4654	776.2657
799.7804	816.4386	856.3454
869.5507	933.5158	973.0709
1049.6492	1060.4682	1066.8488
1069.0717	1092.8513	1127.3470
1137.8429	1144.7548	1147.4243
1153.5023	1168.8293	1188.5059
1205.4991	1223.6885	1241.1228
1246.6428	1249.1039	1252.1726
1255.5478	1273.1294	1293.9269
1300.9051	1320.3927	1322.3260
1351.3157	1369.8160	1394.2410
1408.3203	1443.2033	1448.2006
1463.2477	1477.4357	1487.6473
1511.0343	1519.7621	1520.9757
1528.0618	1531.4689	1536.8607
1542.6862	1645.4331	1652.1379
3054.2805	3059.8179	3061.7033
3098.4805	3105.2765	3105.6601
3127.0198	3137.5264	3145.5719
3167.8440	3199.4160	3222.5203
3327.3194	3344.7300	3348.9155

C-Bmim-PF6

P	0.000000	0.000000	0.000000
N	0.000000	0.000000	4.280100
C	1.101072	0.000000	3.532654
N	1.749513	1.147364	3.711379
C	1.043635	1.909395	4.617210
C	-0.054657	1.191044	4.971603
H	1.376066	2.889892	4.919219
H	-0.866667	1.425984	5.641078
H	1.354056	-0.753294	2.799895
C	2.930212	1.556553	2.953427
H	2.872049	2.631198	2.779295
H	3.838260	1.313189	3.508925
H	2.910256	1.046547	1.988931
C	-1.070186	-1.004696	4.199073
H	-0.671779	-1.839495	3.618927
H	-1.274004	-1.348806	5.217318
C	-2.308710	-0.431754	3.511760
H	-2.000137	-0.022875	2.545510
H	-2.721175	0.390344	4.112690
C	-3.383732	-1.498037	3.284484
H	-2.954983	-2.315371	2.691390
H	-4.168913	-1.051925	2.664783
C	-4.003109	-2.052540	4.569056
H	-3.279762	-2.619779	5.167314

H	-4.398065	-1.244426	5.197549
H	-4.831992	-2.730163	4.340551
F	1.620960	-0.059227	0.352796
F	0.261095	1.168708	-1.081326
F	0.186841	-1.147889	-1.118160
F	-1.601707	0.051464	-0.240530
F	-0.153881	1.131630	1.206539
F	-0.225783	-1.176311	1.163065

12.4086	23.2212	24.7906
35.9408	48.4829	64.4506
68.4948	76.1625	89.9347
120.5067	136.7117	213.5185
231.8388	281.5991	288.1203
292.9492	296.8378	308.8814
357.3229	422.5609	443.5078
445.3253	446.9139	451.9971
533.2437	534.2095	537.2620
557.0099	563.8114	634.7047
644.8216	676.9883	710.7068
747.0933	750.9376	777.2834
808.9460	860.1011	872.1006
878.0748	887.6488	888.5233
907.3145	975.9333	1002.0893
1058.6034	1063.1107	1104.5647
1128.1616	1141.0907	1144.8075
1167.5587	1173.6932	1214.5340
1251.1710	1309.6127	1323.2143
1353.2622	1368.9135	1401.9884
1404.6677	1434.0887	1449.0205
1456.2849	1488.1578	1493.1544
1517.3190	1521.3163	1524.5662
1531.9201	1533.1928	1536.1676
1538.8289	1641.1654	1653.4527
3054.6911	3060.3790	3076.4921
3101.0415	3106.8390	3111.5784
3133.3679	3140.9191	3156.6853
3179.9726	3197.2893	3211.1959
3317.5534	3322.6743	3341.2591

D-n-Butanol

C	0.000000	0.000000	0.000000
C	0.000000	0.000000	1.525432
C	1.408438	0.000000	2.114249
C	1.437439	0.000000	3.637324
O	0.831559	-1.145123	4.211329
H	1.297620	-1.928068	3.908987
H	0.871097	0.848964	4.028092
H	2.472142	0.103640	3.992833
H	1.967016	-0.870828	1.741090
H	1.959118	0.881527	1.760684
H	-0.549333	-0.868656	1.900376

H	-0.539647	0.880895	1.893827
H	0.504773	-0.886907	-0.396551
H	-1.017301	0.006779	-0.400199
H	0.518587	0.878170	-0.397796

83.3902	145.6122	240.8250
252.0669	315.1270	348.7679
534.4322	753.0400	846.1837
864.4555	974.0008	981.8150
1063.5126	1105.5167	1122.3218
1155.2726	1239.3557	1294.4003
1339.7672	1353.0592	1394.7320
1425.9711	1434.2570	1438.1927
1499.7208	1514.0478	1522.5365
1525.9484	1528.5654	3006.7533
3020.0361	3038.4513	3044.4326
3056.9422	3089.4318	3107.7379
3113.3220	3117.2117	3864.4118

E-Octadecane

C	10.819503	-0.146671	0.544642
C	9.562361	0.519980	-0.018522
C	8.300377	-0.322850	0.174615
C	7.030875	0.346110	-0.355897
C	5.753135	-0.441586	-0.051790
C	4.472679	0.360325	-0.288665
C	3.196505	-0.437080	-0.017515
C	1.916115	0.374621	-0.218974
C	0.639160	-0.432082	0.024283
C	-0.637527	0.403185	-0.087208
C	-1.918315	-0.424775	0.020647
C	-3.196835	0.409099	-0.083258
C	-4.467251	-0.438289	-0.227387
C	-5.732164	0.278917	0.253324
C	-7.025982	-0.356683	-0.255854
C	-8.289204	0.327651	0.267579
C	-9.582418	-0.345092	-0.196033
C	-10.836944	0.303623	0.387790
H	10.628376	-0.566292	1.540121
H	11.657341	0.554653	0.617453
H	11.137010	-0.981612	-0.092462
H	9.411462	1.487763	0.480312
H	9.705103	0.742600	-1.084480
H	8.430236	-1.303892	-0.306562
H	8.174354	-0.522310	1.248638
H	7.117052	0.506650	-1.439851
H	6.947042	1.346897	0.094179
H	5.733585	-1.359312	-0.657198
H	5.774176	-0.768698	0.998037
H	4.457459	0.727446	-1.325586
H	4.485164	1.253965	0.352235
H	3.174312	-1.319155	-0.674028

H	3.222298	-0.822641	1.012469
H	1.930257	1.242881	0.456098
H	1.898045	0.781499	-1.240719
H	0.683685	-0.894794	1.021206
H	0.593765	-1.261597	-0.696436
H	-0.638045	0.934987	-1.049996
H	-0.634429	1.179936	0.691650
H	-1.925512	-0.978534	0.970273
H	-1.916522	-1.184366	-0.775980
H	-3.279207	1.038993	0.815241
H	-3.123832	1.102907	-0.932381
H	-4.584713	-0.736530	-1.278606
H	-4.353076	-1.373630	0.339939
H	-5.700607	1.327114	-0.081116
H	-5.737136	0.310006	1.351712
H	-7.028409	-0.337154	-1.355281
H	-7.045744	-1.418843	0.031643
H	-8.292773	1.381423	-0.047433
H	-8.263828	0.337545	1.367689
H	-9.629065	-0.315279	-1.293871
H	-9.557203	-1.408217	0.080139
H	-10.849812	1.382094	0.188587
H	-10.874349	0.173690	1.476469
H	-11.750876	-0.130612	-0.032772

11.6062	17.3172	25.5617
28.9937	41.9488	56.3556
59.9870	73.2919	81.1435
96.5199	101.1474	113.4075
128.8710	131.9298	137.3766
147.8918	152.4178	158.6427
166.0721	171.2248	210.1566
218.5228	249.6858	251.9066
271.0117	339.8667	358.3587
408.4239	441.5822	470.3655
498.4694	523.1172	534.2871
731.4082	737.2966	739.6819
742.0207	745.8257	756.3896
772.2745	794.6797	820.9070
854.2841	889.1551	916.5935
920.1670	923.8756	960.6966
990.0893	1004.6442	1012.5850
1022.2285	1023.6182	1043.3694
1046.2320	1056.4065	1064.0010
1076.1314	1080.8322	1087.3870
1093.0398	1093.9036	1095.3266
1098.5073	1098.9391	1104.6147
1123.6827	1155.5823	1168.6043
1208.5116	1220.9580	1234.5530
1242.8559	1251.9402	1264.1179
1273.3909	1282.9566	1295.7567

1304.7774	1316.2739	1323.8018
1334.1457	1338.1804	1342.2038
1343.3461	1346.9453	1351.9387
1355.6218	1357.1095	1360.0482
1364.2884	1365.4121	1367.4246
1384.6752	1402.1604	1417.3385
1428.5976	1436.1895	1439.6206
1440.4394	1441.3948	1441.4637
1445.8863	1446.9273	1513.0544
1513.1500	1515.3146	1515.5755
1516.1898	1516.4726	1519.0985
1519.3990	1522.1255	1522.2696
1524.9566	1526.2313	1527.4989
1528.8716	1530.8685	1533.7632
1536.3548	1538.3293	1539.5036
1540.7421	3029.1693	3029.6335
3029.8289	3030.0663	3030.9548
3031.9369	3032.6832	3033.6595
3034.4011	3034.7031	3036.3509
3039.6064	3042.0956	3044.0156
3046.0597	3047.2582	3053.3034
3053.4630	3054.7647	3056.4639
3057.4776	3058.7856	3060.4852
3063.5896	3067.5160	3070.9717
3075.3666	3080.5406	3085.0538
3088.9346	3092.4906	3096.3929
3099.3733	3101.2581	3120.9454
3124.7365	3132.9066	3139.3580

F-n-Octane

C	0.000000	0.000000	0.000000
C	0.000000	0.000000	1.528398
C	1.408165	0.000000	2.124355
C	1.419740	-0.002345	3.653073
C	2.827829	0.002113	4.248656
C	2.838711	-0.007220	5.777230
C	4.246561	-0.005048	6.373964
C	4.245637	-0.011872	7.902257
H	-1.019432	0.001689	-0.401156
H	0.513517	-0.885726	-0.393037
H	0.516058	0.884268	-0.393186
H	-0.547569	-0.878824	1.895836
H	-0.547238	0.879061	1.895652
H	1.957657	-0.878392	1.754937
H	1.956084	0.880493	1.757608
H	0.873693	-0.884389	4.018668
H	0.867487	0.874526	4.021723
H	3.381900	-0.872142	3.876623
H	3.372337	0.886704	3.886974
H	2.285968	0.867680	6.150053
H	2.293296	-0.891117	6.139549
H	4.797533	-0.880292	6.002954

H	4.791218	0.877483	6.011048
H	3.732233	0.872353	8.298825
H	3.728643	-0.897627	8.290707
H	5.264878	-0.015777	8.303789

44.4749	59.9495	79.5780
108.8108	133.5968	158.3386
199.9257	247.2128	249.9631
279.9484	346.5712	480.3880
482.7093	738.1716	742.0095
764.8281	818.7414	895.9397
908.8258	925.0587	978.4395
1028.0344	1041.6756	1051.4005
1082.3038	1092.8215	1095.3579
1119.9635	1174.9690	1219.8146
1250.0340	1266.7450	1297.3723
1320.0000	1337.3778	1342.6749
1358.9427	1361.0961	1370.4763
1411.4100	1435.8480	1440.6790
1443.8388	1445.3616	1514.4710
1514.7877	1519.5890	1520.6101
1525.6418	1529.8032	1529.9140
1531.5058	1536.7109	1539.3807
3028.9776	3030.2973	3031.8736
3037.3085	3045.6553	3046.7960
3054.3518	3055.0284	3055.3185
3058.7699	3068.2001	3079.9758
3089.8712	3096.9885	3128.3649
3128.5510	3132.7105	3133.0722
